# Highly Efficient Red Cabbage Anthocyanin Inserted TiO_2_ Aerogel Nanocomposites for Photocatalytic Reduction of Cr(VI) under Visible Light

**DOI:** 10.3390/nano8110937

**Published:** 2018-11-14

**Authors:** Haiyan Yang, Liang Jiang, Yizhou Li, Guoqing Li, Yepeng Yang, Jiao He, Jiaqiang Wang, Zhiying Yan

**Affiliations:** National Center for International Research on Photoelectric and Energy Materials, Yunnan Provincial Collaborative Innovation Center of Green Chemistry for Lignite Energy, Yunnan Province Engineering Research Center of Photocatalytic Treatment of Industrial Wastewater, The Universities’ Center for Photocatalytic Treatment of Pollutants in Yunnan Province, School of Chemical Sciences & Technology, Yunnan University, Kunming 650091, China; haiyan2013new@163.com (H.Y.); liangjiang_ynu@163.com (L.J.); 13698785055@163.com (Y.L.); 18818278290@163.com (G.L.); mondaysunday1234@163.com (Y.Y.); hejiao@ynu.edu.cn (J.H.); zhyyan@ynu.edu.cn (Z.Y.)

**Keywords:** Red cabbage pigment, anthocyanin, TiO_2_ aerogel, photocatalytic activity

## Abstract

In sharp contrast to conventional photosensitization methods in which the organic pigments were often adsorbed, herein we present a study on natural vegetable pigment inserted TiO_2_ aerogel nanocomposites and we directly use red cabbage anthocyanin (RCP) as a structure-directing agent. It was found that pure TiO_2_ aerogel nanocomposite did not exhibit any meaningful activity for photocatalytic reduction of Cr(VI). However, the photocatalytic reduction activity was greatly improved by the RCP inserted TiO_2_ aerogel nanocomposites under visible-light irradiation, which was approximately 2- and 12.3-fold higher than that of TiO_2_ aerogel conventionally photosensitized by RCP and pure TiO_2_ aerogel nanocomposites, respectively. It also exhibited good stability and could be reused at least three times without losing a significant amount of its activity.

## 1. Introduction

Photosensitization of TiO_2_ nanocomposite by organic pigments is a convenient way to develop visible-light efficient photocatalysts since the adsorbed organic pigment molecules are responsible for absorbing the visible light [1,2,3]. Compared with artificial pigments, the natural pigment is more easily available, less costly, less toxic and more environmentally friendly. Currently, natural vegetable pigments have garnered a lot of attention by the scientific community for their nontoxicity, large availability and low cost. For example, natural pigment extracted from blue pea was used to synthesize dye-sensitized TiO_2_ solar cells [4]. The dyes extracted from basella alba rubra [5] and natural apocarotenoids extracted from the achiote’s seeds [6] have been found to be suitable for use as a sensitizer in solar cells. Among the large variety of natural vegetable pigments, red cabbage (*Brassica oleracea* L. var. *capitata* L.) pigment has been explored due to the fact that it contains amounts of anthocyanins and exhibits a wide spectrum of light over a very broad pH range [7,8,9], which is widely used to color various types of food, candies, chewing gum, and yoghurts [10]. Actually, red cabbage anthocyanin (RCP) has been used as a sensitizer in TiO_2_ dye-sensitized solar cells [11]. However, these pigments attached to the TiO_2_ are not thermally or photochemically stable since they are still the same as the original ones. Recently, we have successfully used commercial synthetic dyes to synthesize mesoporous TiO_2_ nanocomposites with good visible-light photocatalytic activity [12,13,14]. In this framework, it is worthwhile to explore stable natural vegetable pigment inspired TiO_2_ nanocomposites with great visible-light photocatalytic activity.

Aerogels have recently attracted great attention in various applications for their high surface area/low-density materials consisting of open and highly porous structure [15,16,17]. Typically, aerogels are prepared by sol–gel method involving hydrolysis and condensation of metal alkoxides, followed by gelation and supercritical conditions with organic solvent [18]. The applications of aerogels such as TiO_2_ and transition metal oxide/silica aerogels in photocatalysis including photodegradation and hydrogen production have been explored [19]. Fast and easy transfer of electron in aerogel due to its high porosity and 3D network encouraged us to synthesize natural vegetable pigment inspired TiO_2_ aerogel [20,21].

Cr(VI) is highly toxic and carcinogenic, and is a common contaminant in wastewater which is used in many industrial production process such as electroplating, leather tanning, metallurgy, paint making [22,23,24]. Photocatalytic reduction processes is an advantageous and useful technique for removal of Cr(VI) [24]. A number of photocatalysts such as Ag/TiO_2_, [25] Cu/TiO_2_, [26] CdS-TiO_2_ [27], Rhodamine B-sensitized TiO_2_ [26], and AgBr/B-doped reduced graphene oxide (RGO) aerogels [28] have been developed for photocatalytic reduction of Cr(VI). Moreover, Wang et al. [29] reported that low molecular weight carboxylic acids can enhance the photoreduction of Cr(VI) with neat anatase TiO_2_. Nevertheless, little attention has been paid to the application of anatase TiO_2_ aerogel nanocomposites, let alone the natural vegetable pigment inserted TiO_2_ aerogel nanocomposites in photocatalytic reduction of Cr(VI). In this work, we provide a new strategy to prepare TiO_2_ aerogel nanocomposites by using red cabbage anthocyanin as a structure-directing agent, rather than external adsorbing after the formation of the TiO_2_ aerogel nanocomposites. The photocatalytic activity of these TiO_2_ aerogel nanocomposites was evaluated by the photocatalytic reduction of Cr(VI) under visible-light irradiation.

## 2. Materials and Methods

### 2.1. Synthesis

The red cabbage anthocyanin was obtained from Yunnan Tonghai Yang Natural Products Co., LTD (Yuxi, China). Titanium isopropoxide was obtained from Aladdin Industrial Inc. (Shanghai, China).

Red cabbage anthocyanin inserted TiO_2_ aerogel nanocomposites: Red cabbage anthocyanin inserted TiO_2_ aerogel nanocomposites were synthesized with a process modified from References [13,16]. A varying amount of red cabbage anthocyanin was dissolved in methanol (50 mL), titanium isopropoxide (15 mL), nitric acid (0.1 mL), and deionized water (4 mL), and was mixed and stirred for 30 min. The obtained gel was kept for aging at room temperature (24 h). The final mixture was then transferred into a standard autoclave (Parr 4843) under nitrogen atmosphere and kept at 245 °C for 1.5 h. Then the pressure was quickly released by venting of solvent vapour. The sample was flushed again with nitrogen for 15 min and allowed to cool down in nitrogen to room temperature. The weight ratios of RCP in samples were 6%, 9%, 12%, 15%, and 18%, respectively, which were labelled as RCP-AG(X) where X = 6%, 9%, 12%, 15%, and 18%. For comparison, without adding RCP, the obtained pure TiO_2_ aerogel was denoted as AG.

Conventionally sensitized TiO_2_ aerogel nanocomposites**:** 1.3 g AG was soaked in the RCP solution at room temperature for one day. Finally, the solid was filtered out, washed with deionized water and then dried in air at 90 °C. The obtained nanocomposites were denoted as S-AG (conventionally sensitized TiO_2_ aerogel nanocomposite).

### 2.2. Characterizations

X-ray powder diffraction (XRD) experiments were conducted on a Rigaku TTRAX III spectrometer with Cu Kα radiation (Rigaku Co., Tokyo, Japan). The data were recorded in the 2θ range 10–90° at a scan rate of 10°/min. The Brunauer–Emmett–Teller (BET) surface areas were measured by the nitrogen adsorption/desorption measurements using a Micromeritics Tristar II Surface Area and Porosity Analyzer (Micromeritics, Norcross, GA, USA). Before the test, the samples were degassed in vacuum at 90 °C for 4 h. Scanning electron microscopy (SEM) was taken on a FEI Quanta 200 ESEM scanning electron microscope (FEI, Hillsboro, OR, USA) operated at an accelerating voltage of 10 kV. High-resolution transmission electron microscopy (HRTEM) imagines were carried out on a JEM-2100 (Japan Electron Optics Laboratory CO., Ltd., Tokyo, Japan) operated at 200 kV. UV–VIS diffuse reflectance spectra were measured on a Shimadzu UV-2401PC photometer (Shimadzu Corp., Kyoto, Japan) over the range from 200 to 800 nm at room temperature. X-ray photoelectron spectroscopy (XPS) measurements were carried out on a PHI5500ESCA (Thermo Fisher Scientific Inc., Waltham, MA, USA) analyzer with 200 W Mg Kα radiation. The base pressure was about ~10^−7^ Pa and the binding energies were referenced to the C1s line at 284.8 eV from adventitious carbon. Fourier transform infrared (FTIR) spectra were obtained on a Thermo Nicolet 8700 instrument (Thermo Fisher Scientific, Waltham, MA, USA).

### 2.3. Photocatalytic Activity

The photocatalytic reduction was carried out in a quartz photoreactor containing 50 mL 15 ppm Cr(VI) aqueous solution and 50 mg of photocatalyst with 40 µmol/L formic acid. The suspensions were homogenized in the dark for 3 h to attain adsorption–desorption equilibrium. The suspensions were irradiated by 350 W Xe lamp with a coloured glass filter (>420 nm), utilized for the purpose of allowing only visible-light radiation. The solutions were stirred during the reaction process with a magnetic stirrer. During illumination, about 5 mL of suspension was filtered to separate the catalyst. The Cr(VI) content was determined at 540 nm by a spectrophotometer (UV722). The experiments were conducted in three parallel experiments and the error bars were added in the figures.

## 3. Results

### 3.1. Characterizations

**Figure 1:**
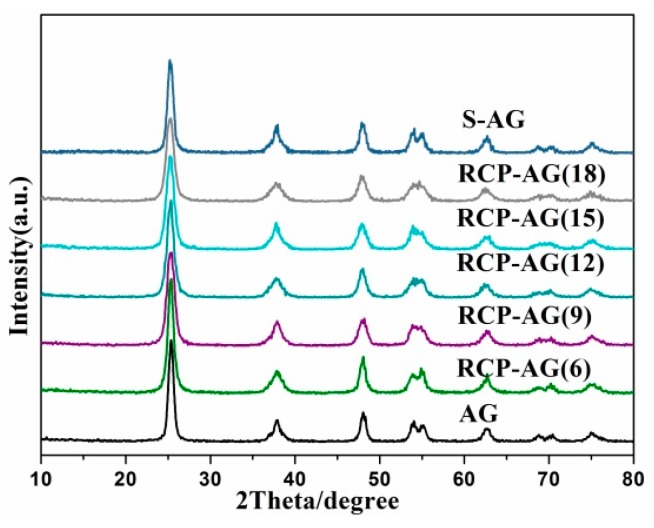
XRD patterns of the samples.

The XRD pattern in Figure 1 reveals that these samples were highly crystallized, suggesting a pure anatase phase. In compared with AG, no other diffraction appeared and the peaks did not shift in S-AG and TiO_2_ aerogel templated by red cabbage anthocyanin, meaning that the addition of RCP would not change the anatase phase of TiO_2_ aerogel [30]. The crystalline size was calculated by using the Scherrer equation. According to (101) diffraction, the crystalline size of AG, RCP-AG(6), RCP-AG(9), RCP-AG(12), RCP-AG(15), and RCP-AG(18) S-AG were 14.4, 10.4, 7.9, 7.8, 8.4, 7.7, and 10.7 nm, respectively. Obviously, the crystalline size became smaller which is due to the increase in RCP concentration from 6 to 18%.

**Figure 2:**
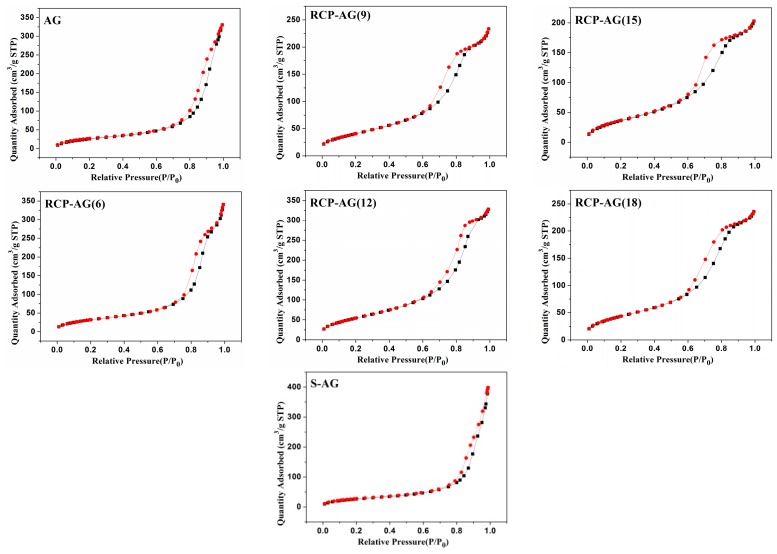
N_2_ adsorption/desorption isotherm of samples.

The N_2_ adsorption/desorption isotherms of the TiO_2_ aerogel materials are similar, as shown in Figure 2. These samples exhibited typical isotherm of type IV having inflection around P/P_0_ = 0.6–0.9, which was the characteristic of mesoporous structure. The specific BET surface areas, pore volumes, pore sizes of samples are described in Table 1. The samples of AG, S-AG, RCP-AG(6), RCP-AG(9), RCP-AG(12), RCP-AG(15), and RCP-AG(18) exhibited surface areas of 101 m^2^/g, 100 m^2^/g, 122 m^2^/g, 149 m^2^/g, 202 m^2^/g, 141 m^2^/g, and 164 m^2^/g, respectively. It is interesting to mention that the S_BET_ increased by using RCP as template. However, when the weight ratios of RCP increased from 12 to 15%, the surface area was decreased from 202 to 141 m^2^/g. At a relative large weight ratio of RCP, the aggregation of TiO_2_ and compact structure were more significant and thus resulted in the surface area being decreased, which was in good agreement with mesoporous TiO_2_ templated by other traditional templates [31,32]. Furthermore, the pore sizes of the synthesized TiO_2_ aerogels displayed the mesoporous region between 7 and 16 nm, which were much bigger than normal mesoporous TiO_2_ [12,13]. However, the pore size significantly decreased when the weight ratios of RCP increased from 9 to 18%. The pores in those samples were filled with some of the compounds which were decomposed from template during the heating under nitrogen atmosphere and thus the pore sizes decreased.

**Figure 3:**
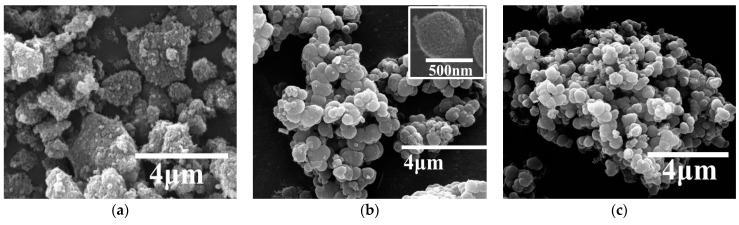
Surface images of (**a**) AG, (**b**) RCP-AG(15), and (**c**) RCP-AG(18).

**Figure 4:**
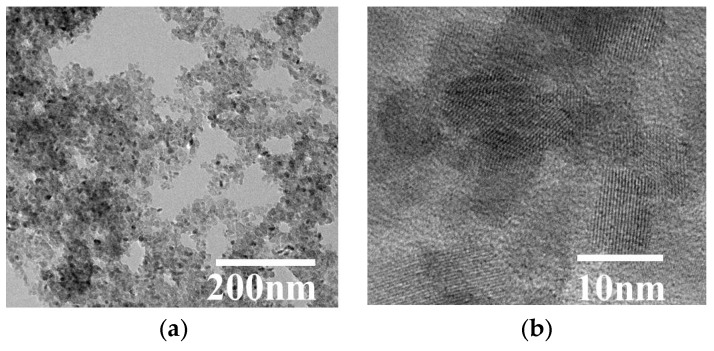
(**a**) The TEM image and (**b**) HRTEM image of RCP-AG(15).

Figure 3a reveals the SEM image of typical TiO_2_ aerogels. It is seen that the TiO_2_ aerogel was highly porous and had spherical-shaped particles. RCP-AG(15) and RCP-AG(18) exhibited the fluffy and irregular shape aggregates (Figure 3b,c). The crystallinity of RCP-AG(15) was observed from the TEM images (Figure 4). TEM measurement illuminates that the size of a single RCP-AG(15) particle was about 9 nm in diameter, which was also in agreement with XRD measurement. High-resolution TEM reveals a lattice spacing of 0.33 nm corresponding to the adjacent (101) crystallographic planes of titania anatase phase [33].

**Figure 5:**
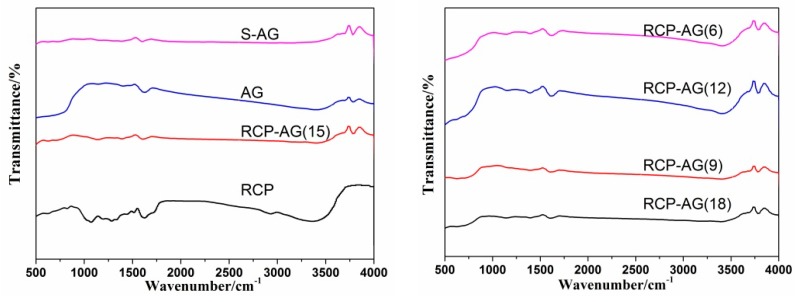
FTIR spectra of samples.

Figure 5 presents FTIR spectra of samples between 500 and 4000 cm^−1^. The two broad peaks at 1620 and 3410 cm^−1^ can be ascribed to the surface adsorbed water and hydroxyl groups [34,35]. One band at 3700–3780 cm^−1^ is characterized by the stretching vibrations of the Ti^4+^-OH surface hydroxyl groups [36]. The peaks at 1390 cm^−1^ and 1400 cm^−1^ assign to -COO^−^ bending vibrations [2], which were usually considered to be photoactive sites [37,38]. However, it is worth noting that the peaks at 2930 and 1070 cm^−1^, which is the characteristic vibration of RCP, are due to -CH_2_- [39] and C-O stretching [40], respectively, and was not found in RCP-AG (15). This confirms that RCP was decomposed during the heating under nitrogen atmosphere.

**Figure 6:**
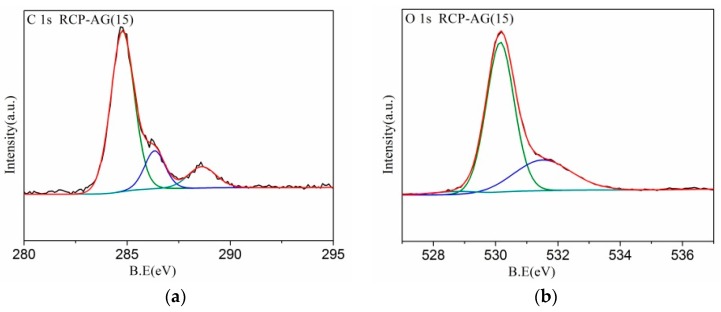

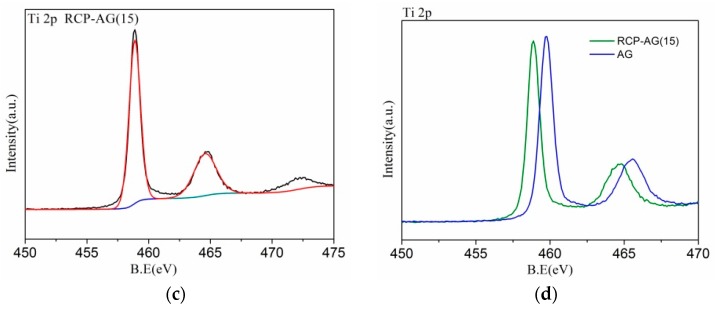
XPS survey spectra of RCP-AG(15) (**a**) C 1s, (**b**) O 1s, (**c**) Ti 2p; XPS survey spectra of AG, and RCP-AG(15) (**d**) Ti 2p.

Table 2 displays the elements of AG and RCP-AG(15) calculated from the survey XPS analysis. There were large amounts of O and C elements on AG, which was probably due to incomplete decomposition of the starting material under nitrogen atmosphere [41,42]. The percentage of C in RCP-AG(15) was 27.5%, which was higher than AG. As shown in Figure 6, the survey spectra confirm that C, O, and Ti elements existed in RCP-AG(15). The O 1 s spectra of RCP-AG(15) revealed the major peaks at 530.3 eV and 531.9 eV, respectively. The peak at 531.9 eV can be related to surface hydroxyl species (Ti-OH) [43]. The peak locating at 530.3 eV is ascribed to Ti-O-Ti bonds. The O1 s binding energies of RCP-AG(15) exhibited slightly shifted (0.84 eV) to the lower value because of the decrease of oxygen vacancies in the TiO_2_ lattice [43,44]. The Ti 2p spectra was consistent with the typical anatase TiO_2_ [40,45,46]. The C1 s spectra can be fitted to three peaks at 284.8, 286.4, and 288.7 eV. The binding energy of 284.8 eV is attributed to C-C and C-H bonds. The peak at 286.4 eV is a typical peak position for C=O band [47]. The peak at 288.7 eV is attributed to the Ti-O-C [44,45,48,49]. However, the peak of Ti-C bonds (281–282 eV) was not found, which also could be deduced that the C atoms did not substitute O atoms in the TiO_2_ lattice structure [50]. Furthermore, compared with AG (459.74 eV), the Ti 2p spectra of RCP-AG(15) was slightly shifted (0.74 eV) to the lower value, which was ascribed to the strong interaction with C [40].

**Figure 7:**
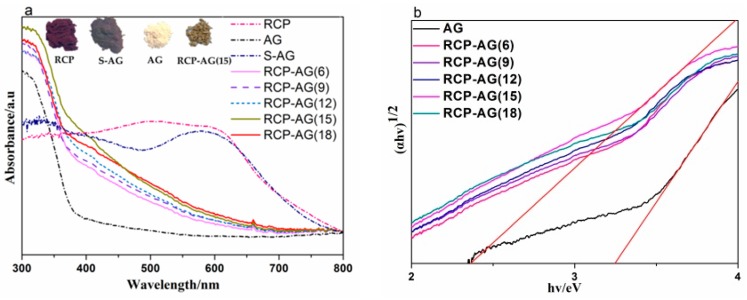
(a) UV-VIS diffuse reflectance spectra of samples; (b) optical band-gap for samples.

The UV-VIS diffraction reflectance spectrum of RCP, AG, and RCP-AG(15) are shown in Figure 7. In comparison with AG, the RCP revealed significant absorption in the visible range. The light absorption of RCP-AG(15) was significantly increased in visible range. The band gaps of the prepared samples were calculated from Tauc plot. The calculated band gap of RCP-AG(15) is 2.36 eV. It indicates that the doped carbon species from RCP can effectively narrow the band gap energy of TiO_2_ aerogel from 3.2 eV (AG) to ~2.36 eV (RCP-AG(15)). Furthermore, RCP-AG(15) exhibited typical red-shift at the edge of the UV and visible-light range and significant absorption occurred in the visible range at ca. 660 nm. By contrast, AG did not reveal significant shift absorption spectra. This may be caused by the doped carbon species, which could narrow the band gap of TiO_2_ to contribute to the red shift and significantly improve the light harvesting ability. These observations indicate why RCP-AG(15) can be potential candidates for performing photocatalytic activity under visible light.

### 3.2. Photocatalytic Tests

To evaluate the potentiality of the prepared samples on photocatalytic reduction of Cr(VI), a set of photocatalytic experiments were performed. In all the experiments, the solution was firstly homogenized during 3 h in darkness in order to achieve the balance of adsorbing–desorbing in the system. Initial tests (with 40 µmol/L formic acid) prove that the Cr(VI) was stable in the absence of catalysts under visible-light irradiation. The adsorption of Cr(VI) by the samples was shown in Table 3. It is evident that the maximum adsorption yields of samples were reached within 60 min. It was found that RCP-AG showed better adsorption activity than pure AG. These results suggest that the adsorption activity of the TiO_2_ templated by RCP may depend on the structure [13,14,41,51,52]. The template of RCP led to a significant increase in surface areas of TiO_2_ aerogel, which would provide more active sites in the reaction [12]. Therefore, TiO_2_ aerogel templated by RCP would provide more active sites in the reaction.

**Figure 8:**
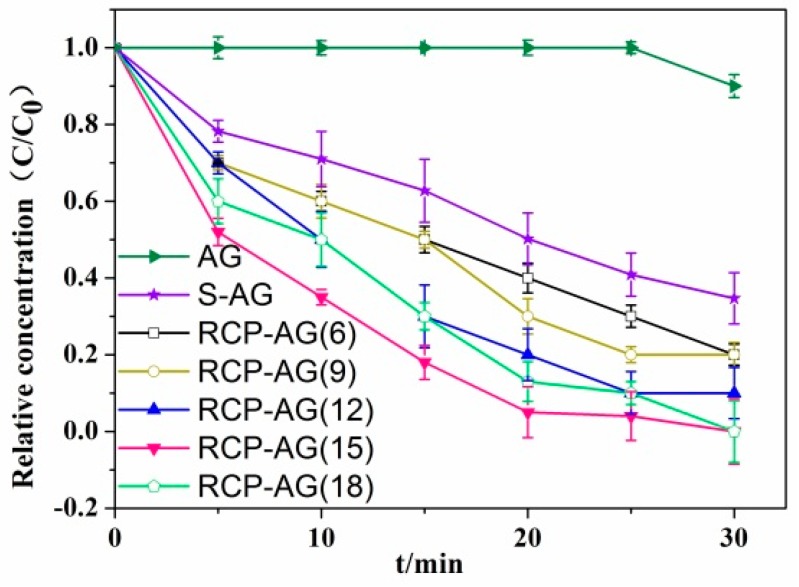
Photocatalytic reduction of Cr(VI) over different samples under visible light with 40 µmol/L formic acid.

Figure 8 reveals the photocatalytic activities for the photocatalytic reduction of Cr(VI) under visible irradiation. Obviously, AG did not exhibit any meaningful activity for photocatalytic reduction of Cr(VI) under visible-light irradiation in 30 min and the photocatalytic reduction activity was greatly improved by the RCP as template. Furthermore, all RCP-AG exhibited significant photocatalytic reduction activity of Cr(VI) and RCP-AG(15) exhibited the maximum photocatalytic yield, which reached 98% in 30 min. This implies that the main photocatalytic activity of RCP-AG(15) was due to the RCP. In particular, the photocatalytic yield for reduction of RCP-AG(15) was approximately 2-fold higher than that of S-AG. The difference between RCP-AG(15) and S-AG was caused by the different bonding strength between RCP and TiO_2_ aerogel. When the RCP are directly used to synthesize TiO_2_ aerogels, the doped carbon species from the decomposed RCP is excited under visible-light irradiation and enhances the charge separation ability [11,53], thus restraining the electron-hole recombination and promoting the photocatalytic activity for Cr(VI).

Figure 8 also exhibited the photocatalytic reduction activity firstly increased and then decreased with the increase of RCP weight ratios from 6 to 18%. In fact, due to the far more intense absorption in the visible region of TiO_2_ aerogel templated by RCP than that of pure TiO_2_ aerogel, it was reasonable that TiO_2_ aerogel templated by RCP showed much better photocatalytic activity than pure TiO_2_ aerogel. The absorption in the visible region trend shown in Figure 7 was different as the weight ratios of RCP and the absorption in the visible region from RCP-AG(15) to RCP-AG(18) had a slightly decline. Thus, the photocatalytic performance of RCP-AG(18) exhibited a decrease.

### 3.3. Effect of pH on the Photocatalytic Reduction of Cr(VI)

**Figure 9:**
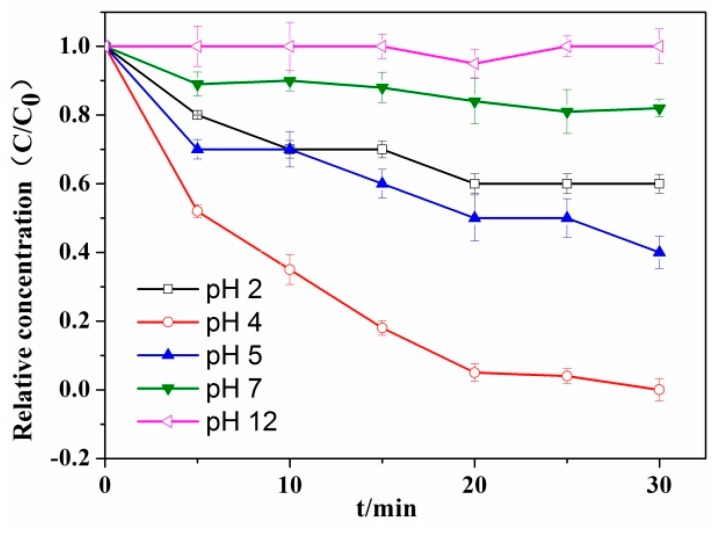
Influence of pH value on the Cr(VI) photocatalytic reduction by RCP-AG(15) under visible light with 40 µmol/L formic acid.

Figure 9 clarifies the influence of pH on the photocatalytic reduction of the RCP-AG(15). The results confirmed that the solution pH had an important influence and the optimal pH value was about 4, whereas the photocatalytic reduction of Cr(VI) was not totally achieved at pH 2. Furthermore, the photocatalytic reduction yield was decreased at the high pH values. In fact, the Cr(VI) ions exist mainly in the form of HCrO_4_^−^, CrO_4_^2−^, or Cr_2_O_7_^2−^ and the RCP-AG(15) is highly protonated at the low pH value, which had a strong affinity toward those anions. Thus, it will enhance the photocatalytic reduction of the Cr(VI) ions. With increasing pH value, the surface of RCP-AG(15) would be less positively charged and the Cr_2_O_7_^2−^ species would be electrostatically repelled by the TiO_2_ surface [50]. That might hinder the photocatalytic reaction of Cr(VI).

### 3.4. Effect of Initial Chromium Concentration on the Photocatalytic Reduction of Cr(VI) by RCP-AG(15)

**Figure 10:**
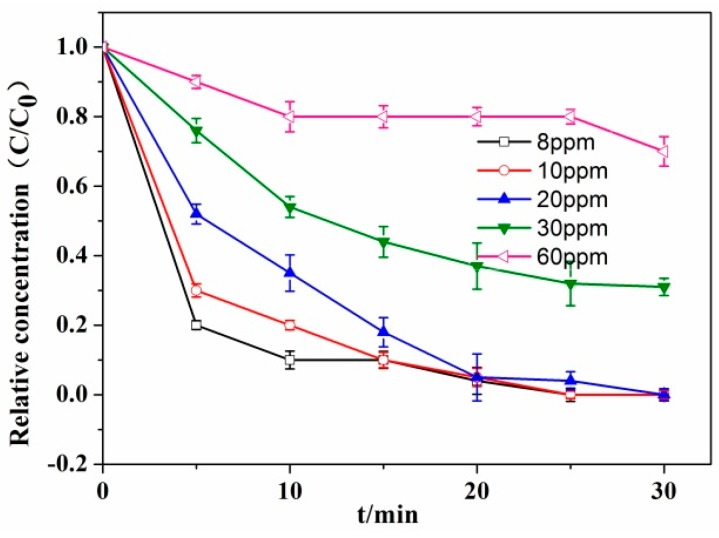
Influence of initial Cr(VI) concentration on its photocatalytic reduction by RCP-AG(15) under visible light with 40 µmol/L formic acid.

The effect of initial Cr(VI) concentration was evaluated by corresponding concentration from 8 to 60 ppm with 40 µmol/L formic acid, using RCP-AG(15). Figure 10 exhibits the photocatalytic reduction activity of Cr(VI) gradually decreasing with the increase of initial concentration. For the low initial concentrations (8 and 10 ppm), the photocatalytic reduction yield of Cr(VI) was 98% after 25 min. However, with 30 ppm Cr(VI), the photocatalytic reduction yield of Cr(VI) was 60% during 30 min. Increasing the concentration to 60 ppm, the photocatalytic reduction yield of Cr(VI) was only 25%, under the same photocatalytic reduction conditions. Similar phenomena had been reported when using CNT/TiO_2_-NH_2_ [54]_,_ TiO_2_-graphene [55], and BiOI [56] as photocatalysts. The increased initial Cr(VI) concentration can enhance the absorption of aqueous solution and prevent the absorption of light in catalyst surface, thus decreasing the photocatalytic reduction activity of Cr(VI).

### 3.5. Recyclability and Stability of Photocatalysts

**Figure 11:**
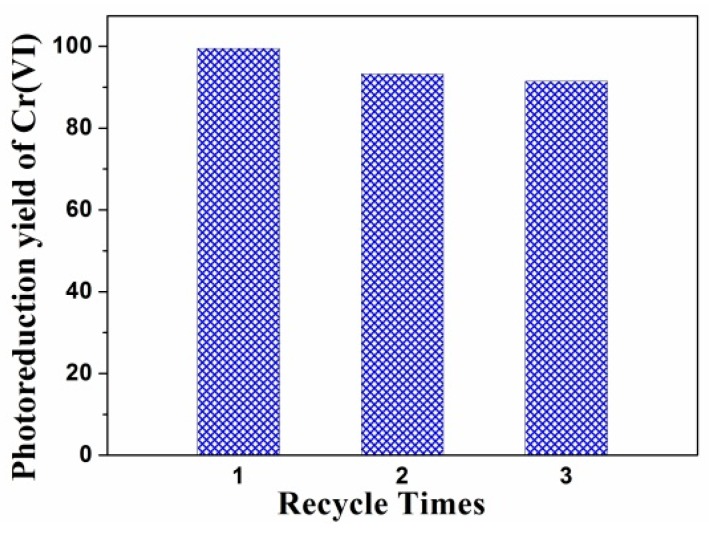
Recycling runs of RCP-AG(15).

**Figure 12:**
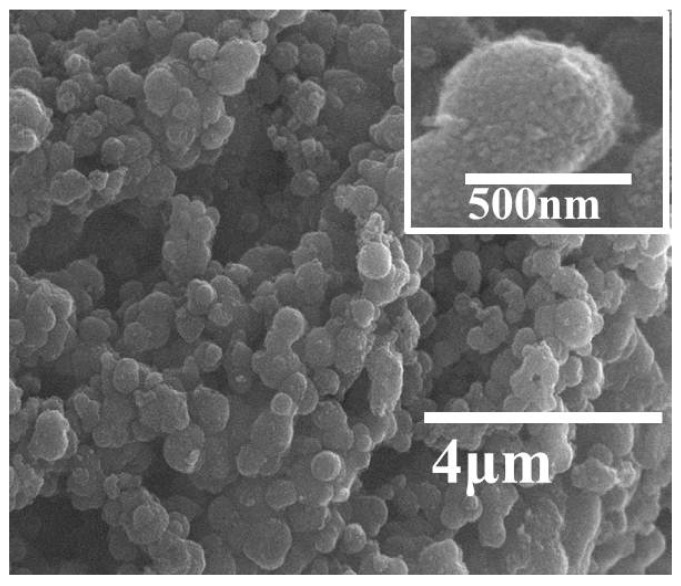
Surface images of the used RCP-AG(15).

**Figure 13:**
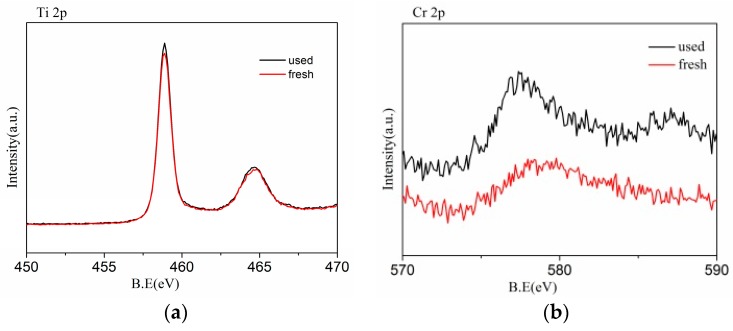
XPS survey spectra of fresh RCP-AG(15) and the used RCP-AG (**a**) Ti 2p, (**b**) Cr 2p.

The recyclability and stability are essential for the practical application of photocatalyst. In this work, the recycling runs of the RCP-AG(15) in photocatalytic reduction of Cr(VI) were evaluated under visible-light irradiation. Figure 11 exhibits the photocatalytic efficiency by the RCP-AG(15) almost unchanged. After three photocatalytic cycles, the photocatalytic reduction yield of Cr(VI) could reach 91%. This indicates that the RCP-AG(15) exhibits a high recyclability for the photocatalytic reduction of Cr(VI). The SEM (Figure 12) and XPS (Figure 13a) of the used RCP-AG(15) had been provided to demonstrate the stability of the photocatalyst. The results prove no significant change in RCP-AG(15) after the photocatalytic reaction process.

### 3.6. Possible Photocatalytic Mechanism

The Cr 2p spectra of fresh RCP-AG(15) and after used RCP-AG(15) were displayed in Figure 13b. The Cr spectrum can be fitted to two peaks of 2p 3/2 and 2p 1/2 orbitals (579.9 eV and 589.1eV, respectively). It is corresponded to the spectra characteristics of Cr(VI). In the fresh RCP-AG(15) spectra, two peaks are not observed in the same position. In the used RCP-AG(15) spectra, a pair of peaks at 577.4 eV and 586.8 eV represented Cr^3+^ 2p 3/2 and Cr^3+^ 2p 1/2, respectively [57]. The presence of a strong signal of Cr(III) is attributable to the photocatalytic reduction of Cr(VI) by RCP-AG(15), which indicates that the catalyst has the photoreduction capability of Cr(VI) and the adsorption ability of Cr(III).

On the basis of the above discussion, it would be interesting to evoke some reasons why the RCP-AG(15) exhibited significant activities for the photocatalytic reduction of Cr(VI) under visible irradiation. The significant photocatalytic activity of RCP-AG(15) was due to the following reasons. The first explanation is that the TiO_2_ aerogel templated by RCP has a high surface area, is highly porous mesoporous and has a network structure. As a result, the structure of RCP-AG may provide more adsorption sites. Secondly, the narrow band gap and the absorption edge of TiO_2_ aerogel templated by RCP was shifted to the visible-light range. The far more intense absorption in the visible region of RCP-TiO_2_ aerogel than that of pure TiO_2_ aerogel would result in the significant photocatalytic activity. Regarding TiO_2_ aerogel, pH can also affect the surface charge of TiO_2_ aerogel and the formation of different Cr(VI) species. In this work, the surface of RCP-AG(15) can be more positively charged and HCrO_4_^−^ was the dominant species at pH 4 [58], which improves the adsorption ability of HCrO_4_^−^ onto the TiO_2_ aerogel surface. Furthermore, the carbon transferred from RCP in TiO_2_ aerogel was beneficial for the photocatalytic reduction of Cr(VI) under visible irradiation. Many investigations [13,49,59] indicate that carbon on the TiO_2_ can promote direct electron transfer and separation. Thus, a possible photocatalytic mechanism for the photocatalytic reduction of Cr(VI) under visible light was proposed. Without light irradiation, Cr(VI) species was adsorbed by RCP-AG. Under visible-light irradiation, electron–hole pairs generated at the surface of RCP-AG. The electrons reduced Cr(VI) to Cr(III), and the holes would oxidize the formic acid [60,61]. The carbon transferred from RCP could suppress the electron and hole recombination by acting as a photosensitizer and enhanced charge separation effect.

## 4. Conclusions

In sharp contrast to conventional photosensitization methods in which red cabbage anthocyanin acted as photosensitizer after adsorbed and were still the same as the original ones, herein we have demonstrated for the first time that red cabbage anthocyanin was directly used as a structure-directing agent without adding any other co-agents during the synthesis of TiO_2_ aerogels and supercritical drying at 245 °C, rather than conventional photosensitization of the TiO_2_ aerogels. These TiO_2_ aerogels with high surface area (202 m^2^·g^−1^) were used for the photocatalytic reduction of Cr(VI) under visible light and exhibited highly efficient photocatalytic yield even when these TiO_2_ aerogels were not modified with any transition metals. Furthermore, this approach is simpler and potentially more stable than TiO_2_ aerogel conventionally photosensitized by RCP. Therefore, we believe that this work provides new insight for the preparation of anatase TiO_2_ aerogel nanocomposites and efficient visible-light photocatalysts in environmental applications, specifically for the treatment of polluted water applications.

## Figures and Tables

**Table 1:** Summary of textural properties and band gaps of the samples.

Samples	SBET (m^2^ g^−1^)	Pore Volume (cm^3^ g^−1^)	Pore Size (nm)	E_g_ (eV)
AG	101	0.6	16	3.24
S-AG	100	0.5	16	-
RCP-AG(6)	122	0.5	15	2.68
RCP-AG(9)	149	0.3	7	2.61
RCP-AG(12)	202	0.5	8	2.49
RCP-AG(15)	141	0.3	8	2.36
RCP-AG(18)	164	0.4	7	2.49

**Table 2:** The surface element composition obtained from XPS analysis.

Samples	Ti 2p (%)	O1s (%)	C1s (%)
AG	20.5	52.2	21.3
RCP-AG (15)	20.9	49.0	27.5

**Table 3:** Summary of adsorption yield in darkness.

Catalyst	Adsorption Yield after 1 h (%)	Adsorption Yield after 3 h (%)
AG	10.5	11.0
S-AG	27.8	28.5
RCP-AG(6)	30.2	30.1
RCP-AG(9)	20.9	21.2
RCP-AG(12)	32.8	33.4
RCP-AG(15)	19.1	20.1
RCP-AG(18)	32.5	32.4
